# Novel ENU-Induced Point Mutation in Scavenger Receptor Class B, Member 1, Results in Liver Specific Loss of SCARB1 Protein

**DOI:** 10.1371/journal.pone.0006521

**Published:** 2009-08-05

**Authors:** Ioannis M. Stylianou, Karen L. Svenson, Sara K. VanOrman, Yanina Langle, John S. Millar, Beverly Paigen, Daniel J. Rader

**Affiliations:** 1 School of Medicine, Institute for Translational Medicine and Therapeutics, University of Pennsylvania, Philadelphia, Pennsylvania, United States of America; 2 The Jackson Laboratory, Bar Harbor, Maine, United States of America; Leiden University Medical Center, Netherlands

## Abstract

Cardiovascular disease (CVD) is the largest cause of premature death in human populations throughout the world. Circulating plasma lipid levels, specifically high levels of LDL or low levels of HDL, are predictive of susceptibility to CVD. The scavenger receptor class B member 1 (SCARB1) is the primary receptor for the selective uptake of HDL cholesterol by liver and steroidogenic tissues. Hepatic SCARB1 influences plasma HDL-cholesterol levels and is vital for reverse cholesterol transport. Here we describe the mapping of a novel *N*-ethyl-*N*-nitrosourea (ENU) induced point mutation in the *Scarb1* gene identified in a C57BL/6J background. The mutation is located in a highly conserved amino acid in the extracellular loop and leads to the conversion of an isoleucine to an asparagine (I179N). Homozygous mutant mice express normal *Scarb1* mRNA levels and are fertile. SCARB1 protein levels are markedly reduced in liver (∼90%), but not in steroidogenic tissues. This leads to ∼70% increased plasma HDL levels due to reduced HDL cholesteryl ester selective uptake. *Pdzk1* knockout mice have liver-specific reduction of SCARB1 protein as does this mutant; however, *in vitro* analysis of the mutation indicates that the regulation of SCARB1 protein in this mutant is independent of PDZK1. This new *Scarb1* model may help further our understanding of post-translational and tissue-specific regulation of SCARB1 that may aid the important clinical goal of raising functional HDL.

## Introduction

Cardiovascular disease (CVD) is the largest cause of premature death in human populations throughout the world. It has long been known that circulating plasma lipid levels, specifically high levels of LDL or low levels of HDL, are predictive of susceptibility to CVD. While compounds exist for lowering LDL, approved treatments for specifically increasing HDL are lacking. The scavenger receptor class B member 1 (SCARB1, also known as SR-B1) is a receptor for HDL that mediates the selective uptake of HDL cholesterol [1] but can also bind to other ligands including unmodified LDL and VLDL [2]–[5]. Knockout *Scarb1^−/−^* mice have elevated HDL cholesterol levels, reduced selective HDL-cholesterol clearance, decreased bile cholesterol concentration and secretion, and thus reduced reverse cholesterol transport (RCT) [6]–[10]. In addition, disruption of *Scarb1* in chow-fed *Apoe*-deficient (*Scarb1*/*Apoe* double KO [dKO] mice) or Western diet-fed LDL receptor-deficient (*Scarb1*/*Ldlr* dKO) mice develop markedly accelerated atherosclerosis [8], [11], [12], demonstrating that elevated HDL and its atheroprotective effects are dissociated when reverse cholesterol transport (RCT) is impaired.

*N*-ethyl-*N*-nitrosourea (ENU) has long been known as the most effective method to introduce heritable mutations in the mouse [13]. With the completion of the mouse genome, ENU has been revisited as a viable route in functional genomics with no less than ten international large-scale ENU screens [14], [15]. Using this approach a mutation identified in a C57BL/6J (B6) screen performed at The Jackson Laboratory (mutant ID: HLB348) [16] and selected based on elevated HDL cholesterol was mapped to the *Scarb1* gene and is described here. Homozygous mutant mice on a ‘pure’ B6 background are fertile and have increased HDL and decreased selective uptake of cholesteryl ester. Furthermore, the mutation causes a 90% reduction of SCARB1 protein levels in the liver but this specific reduction is independent of interactions with PDZK1, which is also known to reduce SCARB1 protein levels specifically in the liver.

## Results

### Identification of the ENU mutant HLB398 and heritability testing

At 8 weeks of age, while consuming standard laboratory chow, a male G3 animal, the founder of the mutant strain discussed in this report, showed an 44% increase in total plasma cholesterol (117 and 168 mg/dL for control and mutant respectively) and a 73% increase in plasma HDL cholesterol (73 and 133 mg/dL for control and mutant respectively) which exceed 2.5 standard deviation units from control means. Repeat testing one week later confirmed these results; therefore the animal was designated mutant HLB398 and entered heritability testing.

**Figure 1:**
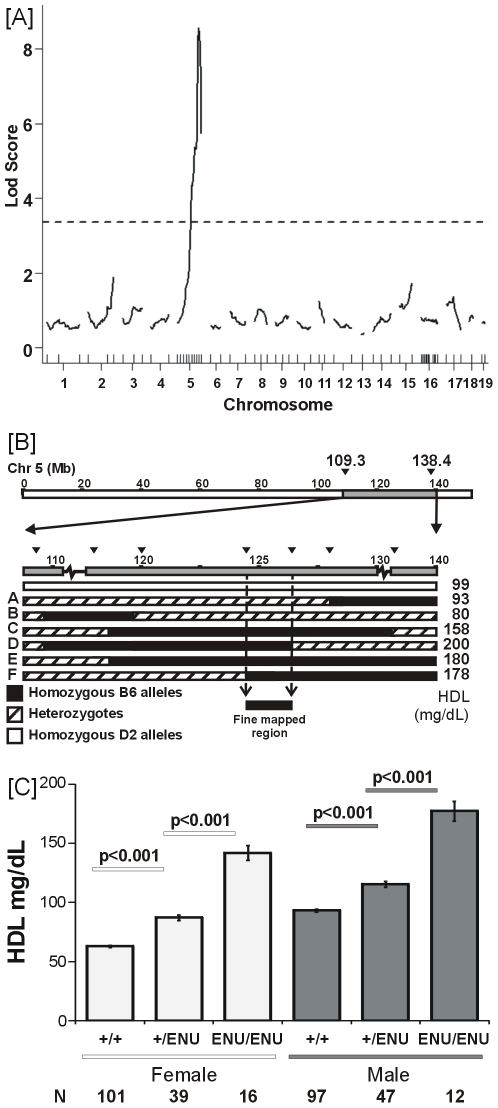
Mapping *ScarbI^179N^*. [A] QTL/mutant mapping analysis (B6-HLB398xD2) for HDL. Dashed line indicates 99% significance threshold after 1000 permutations. Vertical marks above the x-axis indicate location of markers, additional markers were added to Chr 5 after initial analysis indicated chromosome 5 as the only significant locus with B6 alleles giving high HDL levels. [B] Recombinant map and fine mapping on D2 background. Initial QTL mapping located the critical region between 109 and 138 Mb (grey region). Further backcrossing to D2 generated a number of recombinant congenic lines (A–F) narrowing the region to approximately 2 Mb at 124–126 Mb (D5Mit60 - D5Mit214). Triangles indicate locations of markers within the initial fine-mapped region. [C] Distribution of HDL in B6 x D2 mapping population segregating at the *Scarb1* locus showing that the allele effect is additive. HDL mean +/− SEM given for each sex separately and N for each genotype-sex group. Genotypes at the *Sr-b1* locus: +/+ indicates mice with no mutant B6 allele, +/ENU indicates mice with a single mutant B6 allele, and ENU/ENU indicates mice with two B6 mutant alleles. Within sex analysis reveals a significant difference between each genotype group (p<0.001).

A genome-wide scan of the first 77 backcross animals showed that the high HDL ENU mutation mapped to Chr 5 (109 Mb to 138 Mb) with a LOD score of 8.6 (**Figure 1A**). Further mapping, using a combination of intercrosses between backcross animals and continued backcrossing of selected recombinants, yielded 334 total progeny and reduced the genomic location to a 2.1 Mb interval (LOD  =  49.5, D5Mit160 - D5Mit214). This region contains approximately 20 genes, including an obvious candidate *Scarb1* based on its known role in HDL metabolism (**Figure 1B**). The inter-crossing of backcross animals clarified that the effect of the mutation on HDL was additive; female mice had 62, 87, and 142 mg/dL HDL and males were 93, 115 and 177 mg/dL HDL for wildtype, heterozygous and homozygous mutants respectively (t-test p<0.001 between all pairs within sex analysis (**Figure 1C**).

### Fertility

In contrast to *Scarb1^−/^* ^−^ mice, homozygous *Scarb1^I179N^* mice (males and females) had normal fertility. Litter sizes from four homozygous mutant breeding pairs in the original B6 background had a mean of 6.9 (+/−2.4 SD) progeny per litter from ten litters. This is not significantly different from control B6 mice (6.6 progeny per litter +/−0.9 SD). Approximately 1/30 of pups died before weaning age.

Mating of heterozygous mutant mice (B6:D2 mixed backgrounds from the mapping population) yielded homozygous and heterozygous mice in expected frequencies (**Table 1**). Data from 24 backcross litters (B6xD2)F_1_s x D2) segregating at the mutant locus indicates that heterozygous and homozygous D2 alleles are equally likely to occur and that frequency of heterozygous animals is not significantly different between males and females (p = 0.35, 2×2 contingency Fisher's exact test). In addition, eight intercross litters were generated (B6xD2J F_1_s x B6xD2 F_1_s) with parents heterozygous at the mutant locus. The distribution of genotypes in these litters indicate the overall ratio of homozygous D2, heterozygous and homozygous mutant alleles were not significantly different from the expected Hardy-Weinberg ratio (Chi-squared test P = 0.94).

**Table 1:** Observed genotypic ratios at the peak marker from *Scarb1^I179N^* breeding mice.

			*Scarb1* Genotype				
	Litters	Sex	wt/wt	Het	m/m	Total	
Backcross		M	43	43	NA	86	
(B6:D2 F1s) x D2		F	47	35	NA	82	
	24		90	78	NA	168	Total
Intercross		M	8	14	9	31	
(B6:D2 F1s) x (B6:D2 F1s)		F	5	14	9	28	
	9		13	28	18	59	Total

Homozygous D2 alleles shown by ‘wt/wt’; ‘Het’ indicates a single copy of B6 carrying the ENU mutation and a D2 allele, ‘m/m’ indicates homozygous B6 mutants carrying both mutated alleles. ***Backcross population:*** Mice that were heterozygous for the *Scarb1* mutation (B6:D2 derived from the backcross BC1 population) were used for further mapping by backcrossing a second time to D2 (BC2 giving rise to 24 litters. Genotypes of the progeny are not significantly different from the expected frequency. ***Intercross population:*** Nine litters were generated by inter-crossing animals that were heterozygous (from the backcross population BC2) segregating at the *Scarb1* mutation. The ratios are not significantly different from Hardy-Weinberg equilibrium.

### Gene expression

**Figure 2:**
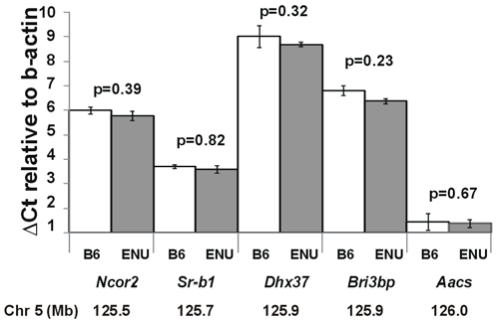
RT-PCR of *Scarb1* and surrounding genes on mouse Chr 5. Liver tissue examined in mutant *Scarb1^I179N^* and B6 control mice (N = 4). Additional genes that mapped in the region and were expressed in the liver according to the SymATLAS database [17] were also tested; *Ncor2*, *Dhx37*, *Bri3bp*, and *Aacs*; but none showed significant differential expression between B6 and the mutant. ΔCt are presented as means with +/−SEM. Correcting for multiple tests using the Bonferroni correction requires p<0.007 for significance at a = 0.05.

Given that *Scarb1* was an obvious candidate, real-time PCR was performed using liver tissue to test expression of *Scarb1* between HLB398 mutant mice and B6 controls. However, no significant difference between the mutant strain relative to control B6 mice was observed. Additional genes that mapped in the region and were expressed in the liver according to the SymATLAS database [17] were tested; *Ncor2*, *Dhx37*, *Bri3bp*, and *Aacs*; but none showed significant differential expression between B6 and the mutant (**Figure 2**). Thus, we concluded that the causal mutation was likely a sequence variant that affected the protein code or translation.

### Sequencing and bioinformatics of *Scarb1*

**Figure 3:**
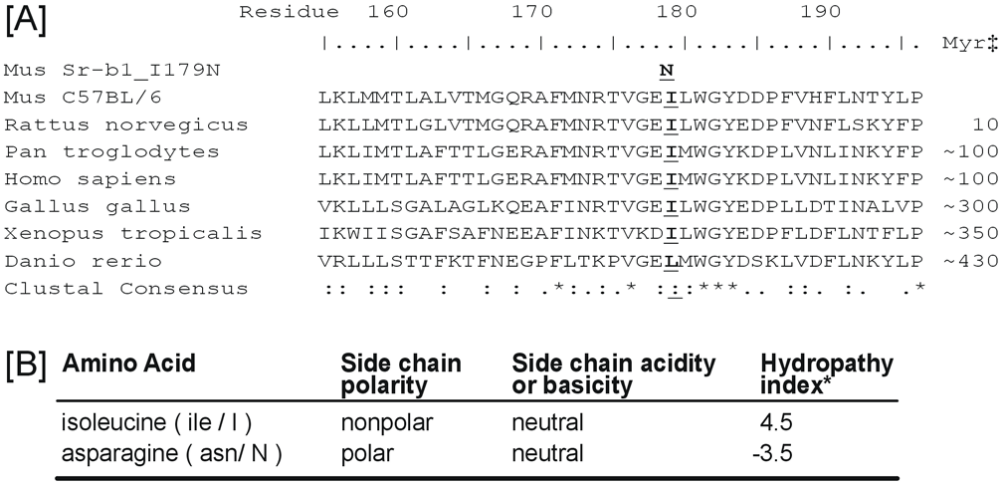
Bioinformatic characterization of the *Scarb1^I179N^* mutation. Exons were sequenced and one single point mutation in *Scarb1* was identified conferring a T to A transversion in the fourth exon at 536 bp from the ATG start site. This causes the 179^th^ amino acid to change form an isoleucine (I) to an asparagine (N) (179N). [A] Conservation: Amino acid sequence for the ENU mutant (Mus_*Scarb1^I179N^*) relative to control un-mutagenized C57BL/6J mice (*Mus*_C57BL/6J) and a number of other species illustrating that the mutation is highly conserved. Sequence alignment was performed using CLUSTALW (www.ebi.ac.uk/clustalw). †Residue number is relative to the mouse sequence. ‡Myr indicates millions of years since a shared ancestor with mouse. CLUSTALW Consensus key; * is a single, fully conserved residue, ‘:’ indicates conservation of strong groups, and ‘.’ shows conservation of weak groups. [B] Properties of the amino acids: isoleucine and asparagine are at opposite ends of the hydropathy spectrum, an index that represents a protein's hydrophobic or hydrophilic properties. The most hydrophobic amino acids are isoleucine (4.5) and valine (4.2). The most hydrophilic are arginine (−4.5) and lysine (−3.9)[53].

Sequencing of exons revealed a single point mutation conferring a T to A transversion in the fourth exon at 536 bp from the ATG start site causing the 179^th^ amino acid to change from an isoleucine (I) to an asparagine (N) (179N). A/T→T/A transversions are the most common type of mutation caused by ENU [14]. Isoleucine 179 is highly conserved in species as distant as *Xenopus* (**Figure 3A**). Although it is possible that a second mutation exists in the mapped (∼2.1 Mb) region causing the observed difference in HDL, the probability of a second mutation leading to a coding change is p<0.002 [18]–[20]. This strain is henceforth designated *Scarb1^I179N^*.

The change from I to N swaps one of the most hydrophobic amino acids (I) with one of the most hydrophilic amino acids (N) (**Figure 3B**) resulting in potentially dramatic physical changes to the protein structure. SCARB1 is heavily *N*-linked glycosylated [21] and thus the introduction of yet another asparagine at 179N could introduce such a post-translational modification. However, due to the lack of a serine or threonine at position 181, *N*-linked glycosylation is not expected to occur.

A search for putative functional sites was performed with the eukaryotic linear motif resource (http://elm.eu.org/) [22]. Using the native mouse B6 sequence, a total of 113 motifs were identified in the SCARB1 amino acid sequence. Of these, only three spanned I179; a forkhead-associated (FHA) Class II domain, a PDZ Class II and a PDZ Class III domain [23]. The FHA Class II and the PDZ Class III domains are not disrupted by the I-to-N change at position 179, but the putative PDZ Class II domain is disrupted. However internal binding to PDZ motifs are rare and while the only known protein to bind with SCARB1 is the PDZ domain containing 1 protein (PDZK1), this protein binds to the C-terminus of SCARB1 [24].

### Western analysis of tissues

**Figure 4:**
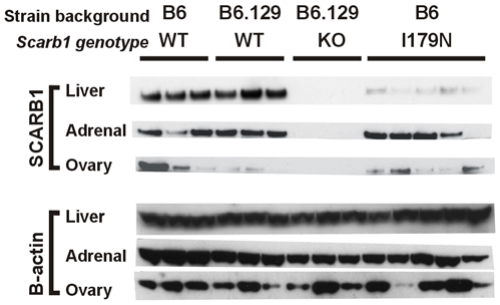
Western analysis of SCARB1 protein levels in tissues. 50 ug of total protein loaded (liver, ovary and adrenals in respective blots) determined by BCA. BACTIN was further used as a loading control.

Western analysis was performed for SCARB1 in female liver, ovaries, and adrenals in *Scarb1^I179N^* mice compared with B6*Scarb1^+/+^* control mice. In liver, *Scarb1^I179N^* mice had just 11.4% of SCARB1 relative to controls (p = 3.2×10^−6^). As reference and additional controls, *Scarb1^−/−^* mice, which are on a mixed B6:129 background, were also compared with control B6.129*Scarb1^+/+^* mice. Interestingly, SCARB1 levels in adrenals and ovaries were unchanged in *Scarb1^I179N^* mice compared to B6 (t-test: adrenals p = 0.95 and ovaries p = 0.39; **Figure 4**). Levels of SCARB1 protein in the ovaries were highly variable for wildtype and mutant mice, as observed previously [9], probably because these experiments did not control for the estrus cycle.

### Serum lipid and lipoprotein analysis

The plasma lipid profile in homozygous *Scarb1^I179N^* mutant mice was analyzed and compared with C57BL/6J controls (hence forth B6*Scarb1^+/+^*) (**Table 2**). For all lipid measurements; homozygous *Scarb1^I179N^* mice were significantly higher than control mice (p<0.01). The relative lipid effects of the *Scarb1^I179N^* mutant compared to controls are comparable to those of *Scarb1^−/−^* mice compared to their controls (**Table 2**).

**Table 2:** Lipid analysis between female *Scarb1^−/−^, Scarb1^I179N^* and control strains.

		mean mg/dL	SE	% change†	
HDL	*Scarb1^I179N^*	93	2.4	171	***
	B6 *Scarb1 ^+/+^*	54	3.2		
	*Scarb1^−/−^*	159	3.5	201	***
	B6.129 *Scarb1 ^+/+^*	79	1.5		
Triglycerides	*Scarb1^I179N^*	121	5.6	222	**
	B6 *Scarb1 ^+/+^*	55	2.2		
	*Scarb1^−/−^*	162	3.4	127	*
	B6.129 *Scarb1 ^+/+^*	128	4.7		
Phospholipid	*Scarb1^I179N^*	205	6.6	119	**
	B6 *Scarb1 ^+/+^*	172	8.2		
	*Scarb1^−/−^*	242	8.0	129	***
	B6.129 *Scarb1 ^+/+^*	187	9.3		
Free Cholesterol	*Scarb1^I179N^*	34	1.3	727	***
	B6 *Scarb1 ^+/+^*	5	1.4		
	*Scarb1^−/−^*	109	1.5	662	***
	B6.129 *Scarb1 ^+/+^*	16	2.2		
Total Cholesterol	*Scarb1^I179N^*	119	4.7	175	***
	B6 *Scarb1 ^+/+^*	68	4.6		
	*Scarb1^−/−^*	184	9.1	190	***
	B6.129 *Scarb1 ^+/+^*	97	5.2		

† % Change indicates change in mutant or KO relative to appropriate control strain. Significance levels *, **, and *** indicate p<0.05, p<0.01, and p<0.0001 respectively. N = 6 animals per group.

**Figure 5:**
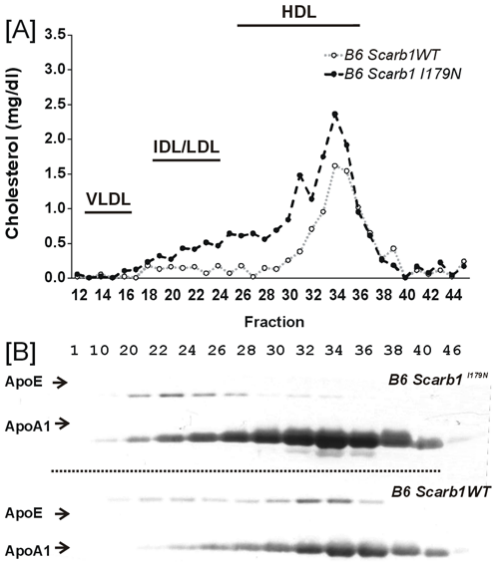
Plasma cholesterol analysis for *Scarb1^I179N^* and control mice. [A] FPLC analysis of plasma lipoproteins: pooled plasma from female mice (N = 6). [B] Western analysis of lipoproteins: 14 ul of respective FPLC fractions and probed for ApoE and ApoAI.

FPLC was performed using pooled plasma (N = 6) from female mice and total cholesterol was assayed for the fractions (**Figure 5A**). *Scarb1^I179N^* mice had a larger HDL peak. Western blot analysis showed that apoA-1 signal peaked at the same location as HDL for the *Scarb1^I179N^* and control mice. *Scarb1^I179N^* mice show an increase in ApoE in the fractions 22–28. These fractions are also accompanied by an increase in ApoA1 when compared to B6 controls and may represent the unusually large species of HDL seen in *Scarb1^−/−^* mice (**Figure 5B**).

### HDL turnover and selective uptake of cholesteryl ester (CE)

**Figure 6:**
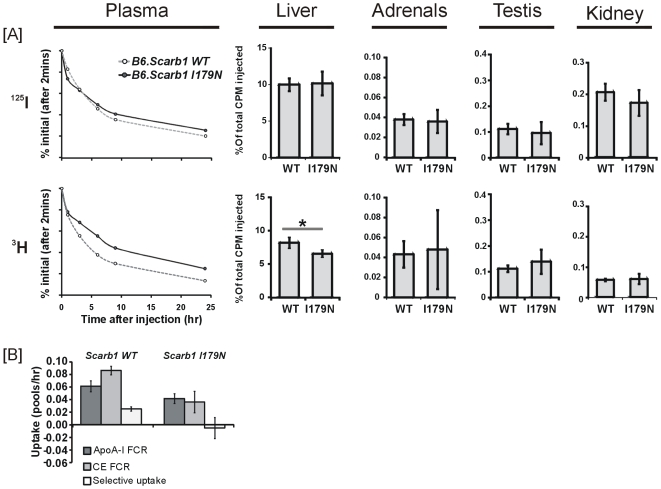
Selective uptake of cholesteryl ester in plasma and liver. [A] Uptake of [^125^I] *upper panel* and [^3^H] *lower panel* in plasma over 24 hrs and in tissues: liver, adrenals, testis and kidney at 24 hrs. *Indicates a significant difference (p<0.01) between *Scarb1^I179^* and control mice in uptake of [^3^H] in liver (means +/−SEM). [B] Uptake in plasma. Data is presented as means +/−SEM. Selective uptake determined from the clearance of double radio-labeled HDL in plasma over 24 hrs. FCR indicates fractional catabolic rate.

Turnover of CE was examined by using double-labeled HDL. HDL protein was labeled with [^125^I]-tyramine cellobiose and cholesteryl ether with [^3^H]. Plasma was monitored after the initial injection for 24hours, after which mice were euthanized and selective uptake measured in tissues. *Scarb1^I179N^* mice showed a reduced clearance rate of CE. In liver tissue, uptake of HDL CE at 24 hours is significantly reduced in mutant *Scarb1^I179N^* mice (50% reduction, p<0.01), (**Figure 6A** **)**, while other tissues (adrenals, kidneys and testis) were unaffected (*data not shown*). In addition, selective uptake as measured in plasma was eliminated in *Scarb1^I179N^* mice (**Figure 6B**). Thus, the decreased SCARB1 protein levels in the liver of *Scarb1^I179N^* mice cause a reduction in HDL CE uptake by the liver, leading to a slower CE clearance from plasma and increased HDL cholesterol.

### *In vitro* analysis of *Scarb1^I179N^*

**Figure 7:**
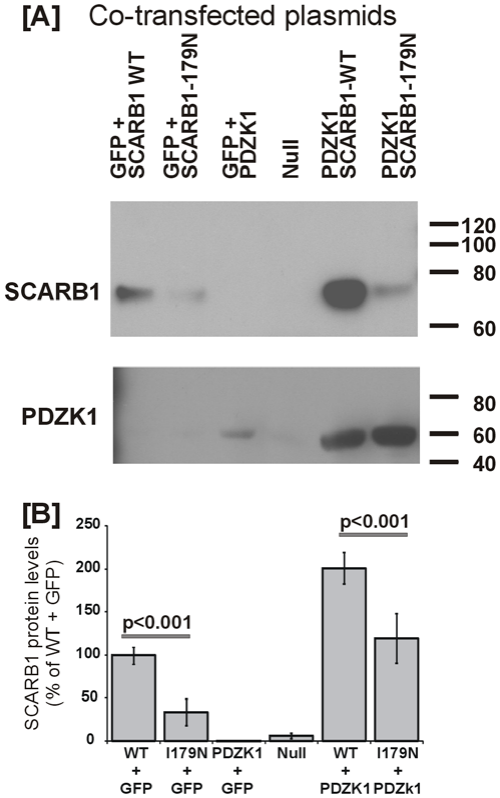
*In vitro* analysis of *Scarb1^I179^*. [A] Co-transfected *Scarb1* wildtype and mutant with *Pdzk1*: Null indicates cells were treated as with other groups but without plasmid added. PDZK1 primary antibody in HuH7 cells was diluted 1∶5,000 to reduced endogenous detection. All other conditions are as described in materials and methods. [B] Means and SE of SCARB1 expression levels from N = 3 wells per group of one experiment, representative of three experiments.

Previous *in vitro* mutant based analysis of SCARB1 amino acids identified several residues that affect HDL binding and N-linked glycosylation sites [21], [25], [26]. However the only residues examined that affect protein levels of SCARB1 are those at the C-terminus that interact with PDZK1. Here, vectors expressing wildtype *Scarb1* and the mutant sequence *Scarb1 ^I179N^* were co-transfected with either GFP or *Pdzk1* expressing vectors (**Figure 7**). Our analysis shows that SCARB1 protein levels are significantly lower in cells transfected with plasmids coding for the mutant sequence relative to wild-type sequence (p<0.001), in agreement with the mutant mouse *in vivo* data.

PDZK1 is known to interact with and specifically affect SCARB1 protein levels in the liver but not other tissues. Co-expressing a vector for *Pdzk1* in cells with a vector for either the *Scarb1* wildtype or mutant, increased SCARB1 protein levels for both variants. However, the increase in SCARB1 protein with the mutant plasmid did not fully restore SCARB1 protein levels to wildtype levels. This was observed in all three cell types; kidney cells (HEK-293), liver cells (HuH7), and smooth muscle cells (MOVAS) (*data not shown*).

Interestingly, in all three cell lines the presence of SCARB1 (wildtype or mutant), significantly increased the amount of PDZK1 (PDZK1+GFP vs. PDZK1+SCARB1 p<0.001) (**Figure 7A**). Transfection efficiencies were determined by GFP expression and RT-PCR for each gene which was not significantly different within each experiment. These results suggest that the I179N mutation causes a liver specific “knockdown” of SCARB1 protein levels that is independent of PDZK1 interaction.

**Figure 8:**
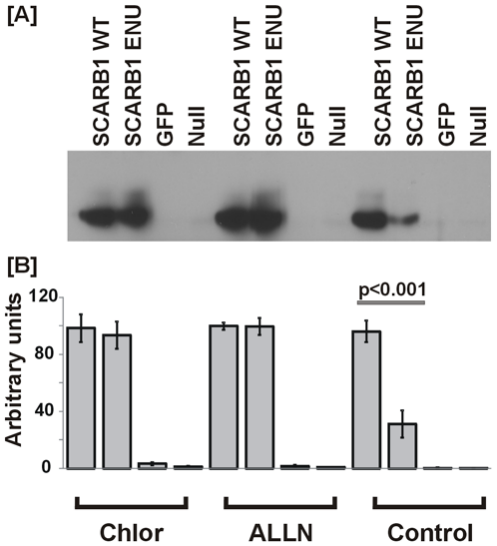
Effects of chloroquine and ALLN on *Scarb1* wildtype and mutant expression. Wild-type (WT), the I179N variant of *Scarb1*, and GFP were transiently expressed using HEK293 cells. 36 h post-transfection, cells were incubated with ±75 µM chloroquine (Chlor, to inhibit lysosomal degradation) or ±100 µM *N*-acetyl-leucinyl-leucinyl-norleucinal (ALLN, to inhibit proteosomal degradation) for 6 hr. [A] Cell samples were collected and SCARB1 proteins were visualized by immunoblotting. Data are representative of three experiments. [B] Means and SE of SCARB1 expression levels from N = 3 wells per group of one experiment. Null indicates no plasmids; cells were treated as with other groups but without plasmid added.

In order to further elucidate the potential mechanism leading to reduced liver SCARB1 protein levels in the mutant, cells were treated with chloroquine to inhibit lysosomal degradation, or with ALLN to inhibit proteosomal degradation. Inhibition of either degradation pathway fully restored SCARB1 protein levels of the mutant to those of wildtype levels (**Figure 8**).

## Discussion

This study identified and characterized a mutant mouse strain with a novel ENU-induced mutation in the *Scarb1* gene (I179N). This mutant presents a phenotype of high plasma circulating HDL in mice with both sexes fertile, in contrast to *Scarb1^−/^* ^−^ female mice, which are infertile. *Scarb1^I179N^* mice have a pure B6 background and thus the 70% increase in HDL is attributed wholly to the single amino acid mutation in SCARB1 that causes a 90% decrease in SCARB1 liver protein levels, leading also to a decrease in selective uptake in livers of mutant mice. Interestingly, SCARB1 protein levels in other tissues examined were not affected. As discussed below, these findings could help in the understanding of SCARB1 protein level regulation. In addition, the B6 background of *Scarb1^I179N^* could yield subtle importance differences that have been previously obscured by quantitative trait loci in other mixed-strain models.

Liver-specific depletion of SCARB1 has previously been reported in *Pdzk1* ^−/−^ mice [27]. *Pdzk1* ^−/−^ mice exhibit similar phenotypes with *Scarb1^I179N^* mutants; SCARB1 protein is reduced by 95% in liver but is unaffected in the steroidogenic tissues (adrenals, gonads), HDL cholesterol is elevated by ∼1.7-fold, and FPLC fractionation of *Pdzk1* ^−/−^ plasma shows increased levels of normally sized HDL particles, more closely resembling *Scarb1^I179N^* profiles shown here rather than *Scarb1^−/−^ mice* [27], [28]. It is therefore possible that the machinery that regulates liver-specific SCARB1 protein levels in *Pdzk1* ^−/−^ mice is also operating in *Scarb1^I179N^*.

PDZ domains usually bind to the C-terminal 3–6 residues of interacting proteins [23], [29]. PDZK1 has four PDZ domains and it has been shown that the first PDZ domain (PDZ1) interacts with the last 3–4 C-terminal amino acids of SCARB1 [30], [31]. Nonetheless, although it has been shown that the SCARB1 C-terminus interacts with PDZ1, the possibility that simultaneous interactions can occur at other locations has not been eliminated. Recently it was shown that over-expression of the PDZ1 domain in *Pdzk1^−/−^* mice did not affect plasma cholesterol, restored only ∼21% of normal SCARB1 protein levels, and the SCARB1 that was restored was located intracellularly rather than on the cell surface [28]. The I179N mutation described here does not occur at the C-terminus; however, PDZ domains do bind to, and interact with, proteins at internal, that is non C-terminal, sequences although such internal binding is rare and poorly understood [23], [32]–[37]. Analysis of PDZ binding motifs revealed a putative Class II PDZ motif, present in mice and conserved in humans, which is not present in the mutant *Scarb1^I179N^* due to the highly hydrophobic isoleucine being replaced by a highly-hydrophilic asparagine. Because of the similarities of *Pdzk1^−/−^* to *Scarb1^I179N^* mice and the questions that remain regarding the PDZK1 regulation of SCARB1, the possibility that the I179N mutation is somehow involved in this interaction deserves further attention. To address this possible interaction the I179N mutation was introduced into vectors for *in vitro* cell culture analysis. The results, which are most prominent in HEK293 kidney cells, demonstrate that the reduction of SCARB1 protein levels is independent of PDZK1. In the absence of PDZK1, *Scarb1^I179N^* expression results in less SCARB1 protein relative to wild-type, and over-expressing PDZK1 does not fully restore mutant SCARB1 protein levels to wild-type levels.

In order to further elucidate the potential mechanism leading to reduced liver SCARB1 protein levels in the mutant, cells were treated with chloroquine to inhibit lysosomal degradation or with ALLN to inhibit proteosomal degradation. Inhibition of either degradation pathway fully restored SCARB1 protein levels of the mutant to those of wildtype levels indicating that the1179N mutant protein is misfolded and degraded. Degradation immediately after protein synthesis is by the proteosomal route, but protein that evades this initial degradation is degraded by the lysosomal pathway after it has reached the plasma membrane. Further studies are needed to elucidate the details of the mechanism and to determine why this degradation or potential differences in turnover are not occurring in other tissues such as the adrenal.

### Strain background of SCARB1 mouse models

It should be noted that HDL and other lipid levels vary greatly among different wildtype inbred strains [38], and the genetic determinants of these differences, called quantitative trait loci (QTL), can be mapped. KO strains on mixed backgrounds such as B6 and 129 will eventually become fixed at all alleles due to inbreeding and drift. Control mice with mixed backgrounds will also become fixed, but for different B6 and 129 alleles than the KO strain. Consequently QTL will confound the phenotypic differences between KO and control mice. HDL levels differ considerably between strains B6 and 129, and these differences are caused by no less than 20 direct or interacting QTL [39], [40]. These QTL are likely to confound characterization of a target gene.

The commonly used *Scarb1^−/−^* knock out model (strain: *Scarb1^tm1Kri^*) [7] was developed using genomic sequence from the 129 strain, and cloned using C57BL/6 (B6) blastocysts. These mice have been the single largest source to our understanding of the function of SCARB1 [41]. However, due to infertility of *Scarb1^−/−^* females and poor breeding on the B6 background, mice are maintained on mixed B6:129 backgrounds. Two additional *Scarb1* mouse models have been developed. *Scarb1^tm1Dhu^*, also described as “SCARB1 att” [9], has a mutation in the promoter that reduces protein levels of SCARB1 in liver by 53% leading to an increase in plasma cholesterol of 50–70%. Protein levels are also reduced in adrenals (83% reduction) and testis. This model was developed using 129/Sv mouse gene sequence cloned into BALB/cByJ blastocysts, and are maintained on a mixed background (129:BALB) [42]. *Scarb1^tm1.1Thh^*, also known as hypomSCARB1-KO^liver^ [43], was developed as a liver-conditional knockout using Cre recombinase *lox*P technology. Mice were developed using 129/Sv ES cells cloned into Alb-Cre transgenic mice having a B6 background (B6.Cg-Tg(Alb-cre)21Mgn/J) and backcrossed six times to B6. While the intention of this line was to generate a liver-specific knock-out, SCARB1 protein was significantly reduced in all tissues to varying degrees, with complete knockdown in liver in the presence of Cre recombinase.

As discussed above, a substitute for *Scarb1* attenuated models could be the *Pdzk1^−/−^* mouse models, since *Pdzk1^−/−^* mice have a 95% liver specific reduction in SCARB1 protein levels and do not have fertility abnormalities. Two *Pdzk1^−/−^* models have been generated thus far, however one is on a pure 129SvEv genetic background (*Pdzk1^ tm1Kr^*) [27], and a second has a mixed B6:129 background (*Pdzk1^tm1Dls^*) [44]. In addition, PDZK1 is known to interact with numerous other membrane associated proteins, including at least one, solute carrier family 22, member 5 (*Slc22a5*, also known as *Octn2*), that affects plasma cholesterol levels [45], [46].

The *Scarb1^I179N^* mutant mice provide a model for studying SCARB1 in a pure B6 background and may help further clarify differences in phenotypes from *Scarb1^−/−^* and *Pdzk1^−/−^* mice. Furthermore, the I179N mutation may further aid our understanding of SCARB1 protein level regulation, trafficking and localization.

## Materials and Methods

### Mice

C57BL/6J (B6) and DBA/2J (D2) strains were obtained from The Jackson Laboratory (Bar Harbor, ME) and maintained on a 12 hour light/12 hour dark cycle. Mice were housed in individually pressurized cages (Thoren Caging Systems, Hazelton, PA), allowed *ad libitum* access to acidified water and food. Mice were weaned onto a standard chow diet based on NIH 31 (LabDiet 5k52, Scott Distributing, Hudson NH). Animal protocols were reviewed and approved by the respective Institutional Animal Care & Use Committees at The Jackson Laboratory, and the University of Pennsylvania.

### ENU mutagenesis and heritability testing

As part of a large-scale, high-throughput phenotyping effort at The Jackson Laboratory, a third-generation ENU mutagenesis scheme was carried out using B6 as the background strain. This program has been described elsewhere [16] and further details can also be found at http://pga.jax.org. Briefly, the third generation derived from mutagenized mice termed “G3” began a 7-week phenotyping protocol at six weeks of age, including blood and plasma analyses, challenge with a high-fat diet [47], evaluation of body composition, and cardiovascular screens. A mouse with an HDL level that was two standard deviations above the mean was selected as a potential mutant and retested. To confirm heritability of the trait, the G3 founder was bred to non-mutagenized B6 mice. Subsequent F_1_ progeny were intercrossed and plasma HDL cholesterol was measured in the F_2_ progeny under the same conditions as the founder. The mutant founding line HLB398 has been designated as Jackson Laboratory stock number JR005495 and is currently available from cryopreserved sperm and embryos. Live mice are available from the authors.

### Mapping

Once the ENU mutant was shown to be heritable, it was mapped by crossing to the D2 strain. D2 was selected as the mapping strain because D2 and B6 mice have similar cholesterol levels on a chow diet (∼60 mg/dL) and because two F_2_ quantitative trait locus (QTL) studies have been performed between B6xD2 [48], [49]. The location of these QTL is informative for the mapping of the ENU mutation.

A course genome-wide scan, was performed using 50 simple sequence-length polymorphic markers [50]. This was followed by additional crosses to generate recombinant mice for Chr 5 which was saturated with 40 markers.

### Phenotyping of mapping population

Blood samples from 8 week old mice fasted for 4 hours in the morning, were collected in tubes containing EDTA, and centrifuged at 9,000 rpm for 5 mins. Plasma was frozen at −2°C until analyzed. Plasma samples were thawed, vortexed and analyzed within a week of being collected. From each plasma sample concentrations of HDL, total cholesterol (TChol) and triglycerides (TG) were measured directly using enzymatic Reagent Kits (#650207, #467825, and #445850 respectively, Beckman Coulter, Inc., Fullerton, CA) used according to manufacturer's recommendations on the Synchron CX Delta System (Beckman Coulter, Inc).

### Gene expression

Gene expression was performed in liver tissue comparing males from homozygous mutant to control B6 mice (N = 4/group). Expression was performed taking the mean of three technical replicates for each of four biological replicates, and relative expression was calculated between target genes and *b-actin* (ΔCt). Tissue collection, RNA extraction, cDNA generation, primer design and all other details are as described previously [51]. Multiple primer pairs for each gene amplifying 100–150 bp fragments with primers spanning exons were tested for relative efficiency using log dilutions of cDNA. Primer pairs were chosen that amplified equivalently (+/−0.25 cycles) over a broad dilution window typically +/−10 fold expression difference, allowing for 100 fold differences (6–7 cycles) to be observed on a linear scale. PCR products of the final primer pairs chosen for RT-PCR were sequenced to confirm amplification of the correct genes.

### Sequencing of *Scarb1*

Exons of *Scarb1* were sequenced for B6, D2 and the ENU mutant strain. DNA was prepared from tails using standard protocols. Cycle sequencing of samples was performed using the BigDye Terminator kit Version 3.1 (Applied Biosystems, Foster City, CA). Sequencing reactions were purified using the XTerminatior kit and run on a 3730xl DNA Analyzer (Applied Biosystems). Sequencing was performed with 2X coverage for all exons and 4X coverage for exon-4 where the SNP was identified. Sequences were analyzed using Sequencher v4.2 (Gene Codes Corporation, Ann Arbor, MI).

### TaqMan SNP assay

A TaqMan SNP assay was developed to distinguish heterozygous from homozygous mutant mice. TaqMan probes; 6FAM: CCACAGGTTCTCAC (MGBNFQ), VIC: CCACAGGATCTCAC (MGBNFQ), Forward primer: TGCTTTTATGAACCGCACAGTTG, Reverse primer: GGAGGTACGTGTTGAGAAAATGCA. Amplification conditions are as recommended by Applied Biosystems for SNP genotyping on an ABI 7500-Real-Time-PCR-System.

### Lipid analysis of the homozygous mutant *Scarb1^I179N^* and control mice

Lipids were analyzed enzymatically using a Cobas Mira-L analyzer (Roche Diagnostic Systems, Inc.

Indianapolis, IN) using the following assays: total cholesterol (Infinity Cholesterol assay, Thermo Electron Corporation, Waltham, MA), HDL (EZ-HDL assay, Trinity Biotech, Co Wicklow, Ireland), and triglycerides (L-Type TG H assay, Wako Pure Chemical Industries Ltd, Osaka, Japan). Pooled plasma (160 uL) from female mice (N = 6) was analyzed with fast protein liquid chromatography (FPLC) gel filtration (Amersham Pharmacia Biotech, Uppsala, Sweden). The cholesterol concentrations in the FPLC fractions were determined with an enzymatic assay (Wako Pure Chemical Industries Ltd).

### Western analysis

Western analysis was performed on livers, adrenals and ovaries from the same female mice used for plasma and FPLC analysis using NuPAGE® 10% Bis-Tris gels (Invitrogen, Carlsbad, CA). Gels were transferred to nitrocellulose membrane (Invitrogen), and blots were visualized with the ECL chemiluminescent detection system (GE Healthcare, Piscataway, NJ). Antibody dilutions were made 1∶5000 (unless otherwise specified) with 5% skim milk.

#### Plasma FPLC fractions

For each fraction, 14 ul was loaded per well. APOE and APOA1 primary antibody: rabbit anti-mouse (Biodesign, Saco, ME), with secondary antibody: HRP goat anti-mouse IgG (Jackson ImmunoResearch Laboratories, West Grove, PA).

#### Tissues

SCARB1 levels were examined in lysates from liver, adrenals, and ovaries loading 50 ug of total protein determined using Pierce bicinchoninic acid assay (BCA) Protein Assay Kit (Thermo Electron Corporation). SCARB1: primary antibody: rabbit polyclonal to mouse SCARB1 (GeneTex Inc., San Antonio, TX) with secondary antibody: donkey anti-rabbit IgG ECL antibody, HRP Conjugated (GE Healthcare). The SCARB1 antibody used was raised to a synthetic peptide (residues 496 to 509: C-SPAAKGTVLQEAKL) which does not include the mutated amino acid and thus is not expected to influence detection.

#### Cell Culture

Cells were scraped off in 1 ml sterile PBS, vortexed and 500 ul was processed with Trizol for RNA. The remaining 500 ul of cells was made to 1X loading buffer and 12 ul total lysate was loaded from each well onto Bis-Tris gels for Western blot analysis as described above. SCARB1 antibody was used as above for tissues. PDZK1: primary antibody affinity purified Sheep Anti-human/mouse-PDZK1 (R&D Systems Minneapolis, MN) used 1∶1,000 with secondary antibody: Anti-Sheep IgG-HRP (R&D systems) 1∶2,000.

### *In vitro* site-directed mutagenesis and cell culture

Mouse *Scarb1* (BC004656) and *Pdzk*1 (BC013512) cones were obtained in pCMVSport6 vectors (OpenBiosytems - Thermo Electron Corporation). A control vector for green fluorescent protein also driven by CMV (pIRES-EGFP) was obtained from Invitrogen. Vectors were cloned and sequenced and the I179N mutation was introduced into *Scarb1* by site directed mutagenesis using QuikChange Site-Directed Mutagenesis Kit (Agilent California, USA), by mutating the 536^th^ base pair from the ATG start site from a T to A as observed in the *Scarb1^I179N^* mutant mouse.

HEK-293, MOVAS (ATCC Manassas, VA) and HuH7 cells (Cellbank, Japan) were obtained and maintained as recommended from the supplier. All cell lines were cultured in Dulbecco's modified Eagle medium (DMEM, Invitrogen) containing 10% fetal bovine serum (Sigma) and 1% antibiotic/antimycotic (Invitrogen). Media for HuH7 cells also contained 0.2 mg/mL G418 (Invitrogen). HEK-293 and HuH7 cells were transfected with Lipofectamine (Invitrogen), while MOVAS cells were transfected with Lipofectamine 2000 (Invitrogen). Cells were plated in12-well plates 24 hrs post transfection. Transfections were performed by co-transfecting an equal amount of plasmid for each gene in triplicate wells for each experiment. Each experiment was itself performed in triplicate. Transfections were allowed to progress for 5 hrs in 500 ul OptiMEM (Invitrogen) then 500 ul 20% FBS DMEM was added to the cells allowing the transfection to progress to 48 hrs.

HEK293 cells were also used for inhibition of degradation pathways. Media was replaced at 36 h post-transfection with media containing either 75 µM chloroquine, 100 µM N-acetyl-leucinyl-leucinyl-norleucinal (ALLN, Sigma), or PBS (controls). After the 6 hour incubation with chloroquine or ALLN, cells were collected and processed as described above Western analysis.

### HDL turnover and selective uptake

HDL was double labeled with [^125^I] on protein and [^3^H] on cholesteryl ether. HDL was iodinated using a modification of the method described previously [52] where tyramine cellobiose was first coupled to HDL prior to iodination. [^125^I]-labeled HDL was subsequently exchange labeled with [^3^H]-cholesteryl ether [52]. Both the [^125^I]-tyramine cellobiose and cholesteryl ether tracers are non-hydrolysable and therefore accumulate in tissues following uptake. Double labeled HDL was injected into the tail veins of 4 hr fasted, 12 wk old male mice. Blood samples were drawn by retro-orbital bleeding at 2 min, 1 hr, 3 hrs, 6 hrs, 9 hrs, and 24 hrs following injection. Mice were sacrificed at 24 hrs and tissues (liver, adrenals, kidneys, and testis) were removed for measurement of tissue uptake.
